# Development of a self-assessment teamwork tool for use by medical and nursing students

**DOI:** 10.1186/s12909-016-0743-9

**Published:** 2016-08-24

**Authors:** Christopher J. Gordon, Christine Jorm, Boaz Shulruf, Jennifer Weller, Jane Currie, Renee Lim, Adam Osomanski

**Affiliations:** 1 Sydney Nursing School, The University of Sydney, Sydney, 2006 NSW Australia; 2 Sydney Medical School, The University of Sydney, Sydney, NSW Australia; 3 Faculty of Medicine, University of New South Wales, Sydney, NSW Australia; 4 Faculty of Medical and Health Sciences, University of Auckland, Auckland, New Zealand; 5 Northern Clinical School, The University of Sydney, Sydney, NSW Australia; 6 Sydney Adventist Hospital Clinical School, The University of Sydney, Sydney, NSW Australia

**Keywords:** Debriefing, Self-assessment, Simulation, Student, Teamwork

## Abstract

**Background:**

Teamwork training is an essential component of health professional student education. A valid and reliable teamwork self-assessment tool could assist students to identify desirable teamwork behaviours with the potential to promote learning about effective teamwork. The aim of this study was to develop and evaluate a self-assessment teamwork tool for health professional students for use in the context of emergency response to a mass casualty.

**Methods:**

The authors modified a previously published teamwork instrument designed for experienced critical care teams for use with medical and nursing students involved in mass casualty simulations. The 17-item questionnaire was administered to students immediately following the simulations. These scores were used to explore the psychometric properties of the tool, using Exploratory and Confirmatory Factor Analysis.

**Results:**

202 (128 medical and 74 nursing) students completed the self-assessment teamwork tool for students. Exploratory factor analysis revealed 2 factors (5 items - Teamwork coordination and communication; 4 items - Information sharing and support) and these were justified with confirmatory factor analysis. Internal consistency was 0.823 for Teamwork coordination and communication, and 0.812 for Information sharing and support.

**Conclusions:**

These data provide evidence to support the validity and reliability of the self-assessment teamwork tool for students This self-assessment tool could be of value to health professional students following team training activities to help them identify the attributes of effective teamwork.

**Electronic supplementary material:**

The online version of this article (doi:10.1186/s12909-016-0743-9) contains supplementary material, which is available to authorized users.

## Background

Teamwork is known to impact on patient care and safety [1] and effective teamwork can improve patient outcomes [2, 3]. Evidence supports the need for ongoing teamwork training to reduce avoidable errors and health care costs [3, 4] and simulation is well-recognised as a teaching method to improve teamwork [5, 6] and patient safety [7, 8].

It has been suggested that organizations could benefit from building a critical mass of staff who have acquired ‘transportable’ teamwork competencies relevant across many settings and teams [9]. Health professionals need to function in dynamic, *ad-hoc* teams with multiple team memberships [10]. Tannenbaum et al. [9] coined the term ‘flash team’, referring to teams that form without members having worked together. These ‘flash teams’ are common in healthcare [11]. Using immersive simulation to teach teamwork in large undergraduate programs presents the dilemma of large numbers of students with limited faculty resources.

Teamwork training has now been established in many undergraduate curricula. However, several issues arise. Firstly, most teamwork measurement tools have been designed for practising clinicians in demanding clinical contexts, rather than for students. Secondly, most published studies have relied on external faculty observing and assessing the team behaviours of students to facilitate feedback in debriefing sessions following the simulation [12–14].

The latter is resource intensive and the high cost of small group simulation and expert debriefing may limit the exposure that most students have to experiential teamwork training. There is some evidence that other approaches that facilitate reflection may be useful. Interprofessional within-team debriefing, using video self-review, has been shown to be as effective as traditional facilitator-led debriefing [15]. At the least, this suggests that simulation participants are able to debrief when facilitators are not experienced or available. Self-debriefing is dependent on participants ability to engage in self-assessment, which is critical to the learning process [16]. A teamwork rating tool with explicit descriptors of the behaviours of effective teamwork could assist this self-assessment.

There are numerous tools that assess teamwork performance, however, these have been developed for practising health professionals and for specific contexts. The Mayo High Performance Teamwork scale which assesses the effectiveness of team training has been used extensively in practicing health professional populations [17]. TeamSTEPPS is a team training intervention which developed a self-report that was shown to have construct validity in multidisciplinary healthcare professionals [18]. A valid teamwork measure for intensive care teams after simulated critical events was developed [19] and then validated later as a self-assessment tool [20]. However, teamwork tools for self-assessment that are designed specifically for health professional *students* are lacking. Furthermore, teamwork tools designed for complex and highly specialised clinical domains such as critical care or the operating room may not be appropriate for health professional students, with their limited clinical knowledge and lack of experience working in healthcare teams. As such, the authors developed a self-assessment teamwork tool, based on the Weller et al. [19, 20] teamwork tool, appropriate for health professional students and in a specific clinical context more relevant to the knowledge and skills of students.

The aim of this study was to develop a self-assessment teamwork tool for medical and nursing students and to explore its reliability and validity.

## Methods

To engage large numbers of students in a stressful clinical environment with time pressures of managing trauma patients, a mass casualty simulation was utilised.

### Participants

Second and fourth year medical students (4 year graduate program) and final year nursing students (2 year graduate program and 3 year undergraduate program) from a large Australian university were invited to participate. Students were recruited via an email providing details about the study. Ethical approval was granted by the University Human Research Ethics Committee (2014/425 and 2014/697) and all students provided written, informed consent prior to commencing the study.

### Instrument development

The 17-item SATTS questionnaire used a 7-point Likert scale ranging from poor (scored as 1), to excellent (scored as 7). Weller et al. [19, 20] provided descriptors for each item, used to assist scoring. See Additional file 1.

The SATTS questionnaire was adapted from the 23-item teamwork tool developed for critical care teams (doctors and nurses) (Weller et al. [19, 20]). The tool has 3 factors with constructs: *leadership and team co-ordination, sharing situational information, and mutual performance monitoring*. In their psychometric analysis of the teamwork tool, Weller et al. [19] found that 20 items were associated with the three constructs, with three items not loading against any factor. For the SATTS, 12 items were retained from Weller et al. tool, including modification of two leadership-specific items in the *leadership and team co-ordination* construct. For these two items, the word ‘leadership’ was removed, as example, ‘*the leader’s plan for treatment was communicated to the team’,* was modified to ‘*a plan for treatment was communicated to the team’* as leadership was considered to be an advanced teamwork attribute [1]. The remaining seven items were deemed too clinically complex for student-led teamwork. Four new simpler items related to teamwork communication and informational sharing were included, as these have been shown to be critical to teamwork [13]. A final item that rated overall teamwork was also included to provide students with a general rating of teamwork, although this has not been included in the analyses. These new items were developed by teamwork experts who considered a range of items and assessed their suitability and relevance for students and the context of the planned simulation activity.

### The simulations

Two full-scale simulation scenarios were constructed with support from expert simulation facilitators. On two separate days, two mixed cohorts (medical and nursing students) were immersed into mass casualty scenarios designed to provide novel and challenging situations that students had not encountered previously in clinical practice. The two simulations were designed to have similar levels of difficulty (patient casualties) and support services (simulation facilitators who were emergency nurses and physicians, and paramedics). Student teams were first responders to events where treatment of numerous casualties was required. The SATTS were administered to students immediately following the simulation activities and they then attended a debriefing session.

#### Simulation activity one

A disaster scene replicating building collapses from the 2011 Christchurch earthquake in New Zealand in which 185 people died was used. The teaching auditorium was constructed of eight disaster clusters each containing four patients over a total space of approximately 600 m^2^. To augment physical and psychological fidelity, video footage of the earthquake was played at the outset and loud sirens were broadcast to imitate emergency services. This had been successfully piloted the previous year with a voluntary cohort of 117 s year medical students [21].

All students were provided a 15-min briefing outlining the scenario, assembled into groups of 4–5 students, and entered the disaster zone. Standardised patients were played by medical students who had injuries marked on their bodies with large adhesive stickers.

Student teams were required to assess, provide first-line treatment and later, triage information to emergency services when they arrived. The scenario unfolded over 50 min and patient observations were added via a large screen as the scenario progressed. An expert simulation facilitator observed four student teams simultaneously and provided limited cues about the patient’s injuries and management required.

#### Simulation activity two

The second scenario consisted of a 21^st^ birthday party where the roof of the hall collapsed falling onto guests below, injuring 28 people. Similar to simulation one, a large teaching hall was used to recreate the disaster scene with physical props including debris (bricks, plaster board), party wear, tables and chairs. Standardised patients who acted as casualties were played by faculty members and health science students with moulage applied to create mock injuries.

At the beginning of the simulation all students watched a pre-recorded short video handover (<90 s) from the scene commander of the incident which included information on the number of casualties and hazards at the scene. Students were allocated to groups of five consisting of four final year nursing students and one final year medical student. Each student team was required to attend to two injured patients. Simulation facilitators were available within the scenario to assist student teams with clinical information related to physical deteriorations and improvements in the casualties’ health status and to ensure safety. The student teams’ main task was to undertake assessment and early interventions to prevent further clinical deterioration of disaster patients. After 30 min, teams were approached by a paramedic and told that they are able to transfer patients to hospital and teams were required to determine which patient to transfer.

### Debriefing sessions

After scenario one, students reassembled into their teams and participated in a 30-min detailed debriefing and feedback with their expert facilitators. The aim of the debriefing was to focus on the students’ experiences and behaviours on teamwork and to highlight components of team interactions. This was followed by a full-cohort debriefing that generalised the simulation experience to the major concepts associated with teamwork and human factors.

At the end of the second simulation activity, students attended a 10-min facilitated debrief which provided an opportunity for students to discuss emotional experiences during the simulation. A whole-group facilitator-led debrief followed in which a panel of emergency specialist experts (physicians and nurses) provided guidance on the teamwork management of the casualty presentations and real-world experience-based perspectives.

### Statistical analysis

All data are presented as means and standard deviations (±SD). Exploratory factor analysis (EFA) and confirmatory factor analysis (CFA) were performed to determine questionnaire factor structure. Factor analysis has been used extensively in questionnaire development to reduce the number of items to composite variables, known as factors. The analysis determines the intrinsic dimensions which are found between the measured variables and latent constructs. Factor analysis provides evidence of construct validity [22, 23]. Exploratory factor analysis is used typically when researchers do not have predetermined expectations of the number of variables in each factor.

Principal Component Analysis, using Principal Axis Factoring with varimax rotation, was used to investigate common variance in the questionnaire. Items with an inter-item correlation less than 0.30 were removed. Kaiser-Meyer-Olkin measure of sampling adequacy was determined with scores greater than 0.5 considered sufficient. Only eigenvalues greater than 1.0 were retained. Any factor coefficient greater than 0.4 was kept for interpretation of the factor structure. Corrected-item total correlation (the degree to which each item correlates with the total score) was performed to identify items that are problematic and need to be revised or discarded. Items with cross factor loadings were removed to improve factor structure.

The recommended ratio of cases to variables is 5:1 if data are normally distributed and 10:1 if not [23]. This study met the criteria for >10:1 [24]. Cronbach alpha was used to determine internal consistency across items. Cronbach alpha of greater than 0.70 was deemed to be acceptable reliability coefficient for internal consistency of the tool. Exploratory factor analysis was performed using SPSS Version 20 (SPSS Inc., Chicago, IL, USA).

In contrast to EFA, CFA is used to test a model assumption of the number of factors and the degree of fit [25]. Confirmatory factor analysis reports several indices that determine the model acceptability. The chi-square goodness of fit test (*χ*^2^) and the goodness of fit index (GFI). The GFI is used to determine differences between observed and predicted covariance matrices and should approach one. The root-mean-square error of approximation (RMSEA) is considered suitable when in the range of 0 to 1. The other indices, the comparative fit index (CFI) and Tucker-Lewis index (TLI) are deemed acceptable when close to 0.95 when RMSEA values are near 0.06 [26]. Confirmatory factor analysis (AMOS Version 22) was undertaken to estimate the model fit of the factor structure as identified by the EFA.

## Results

202 students were enrolled into the study: 128 medical students and 74 nursing students, with 145 students attending the first simulation scenario, and 57 students completing the second scenario. There were more female students (66.2 %) and a minority of students had participated in a previous interprofessional learning experience with other health students (16.8 %).

The authors used several well-agreed criteria to fit the exploratory factor analysis [27]. Two items (item 16: *when faced with a problem, external assistance was sought* and item 17: *situational updates were given when the situation changed*) were removed from the analysis as they had inter-item correlations less than 0.30. Kaiser-Meyer-Olkin measure of sampling adequacy was deemed acceptable at 0.912, greater that 0.5 scores. Bartlett’s test of sphericity was statistical significant (*χ*^2^ (136) = 1425.67, *p* < 0.001). These measures were at an acceptable level to continue with the EFA.

Over 60 % of the cumulative variance was explained within a 2-factor solution. The first factor had an eigenvalue of 4.32, accounting for 48.0 % of the variance, with the second factor having an eigenvalue of 1.10, with a further 12.3 % of the variance.

After removing items that cross-loaded, EFA was repeated and identified a 2-factor structure, consisting of 5 and 4 items, respectively (Table 1). These 2 factors aligned with the constructs *Teamwork coordination and communication*, and *Information sharing and support*, which had been determined from the literature [17, 28]. The correlation between the 2 factors was 0.71. Cronbach alphas were 0.823 and 0.812 for the two factors, respectively.

**Table 1:** Exploratory factor analysis

Item	Factor 1	Factor 2
2. Each team member had a clear role	0.791	*0.230*
6. A plan for treatment was communicated to the team	0.754	*0.275*
5. When team members received instructions they closed the communication loop	0.720	*0.251*
4. Instructions and verbal communications were directed	0.706	*0.194*
1. An overview of the situation was maintained	0.676	*0.211*
15. Suggestions were invited from within the team when problem-solving	*0.151*	0.796
14. Team members offered assistance to one other	*0.277*	0.775
13. Team members sought assistance from each other	*0.362*	0.748
12. Situational information was verbalised	*0.209*	0.646
*3. Instructions were explicit* ^a^	*0.584*	*0.402*
*7. Priorities and orders of actions were communicated to the team* ^a^	*0.745*	*0.390*
*8. Possible future developments or requirements were communicated clearly* ^a^	*0.693*	*0.380*
*9. Questions, input, or requests for clarification were responded to appropriately* ^a^	*0.519*	*0.439*
*10. When expressions of concern were raised and not responded to appropriately, team members persisted in seeking a response, or took action* ^a^	*0.596*	*0.426*
*11. Important clinical actions were verbalised* ^a^	*0.445*	*0.627*

*Key:* Factor 1: Teamwork coordination and communication; Factor 2: Information sharing and support, ^a^ and italicised text indicate cross-loaded items not used in the 2 factors. Principal component analysis with varimax rotation (Kaiser normalisation)

**Fig. 1:**
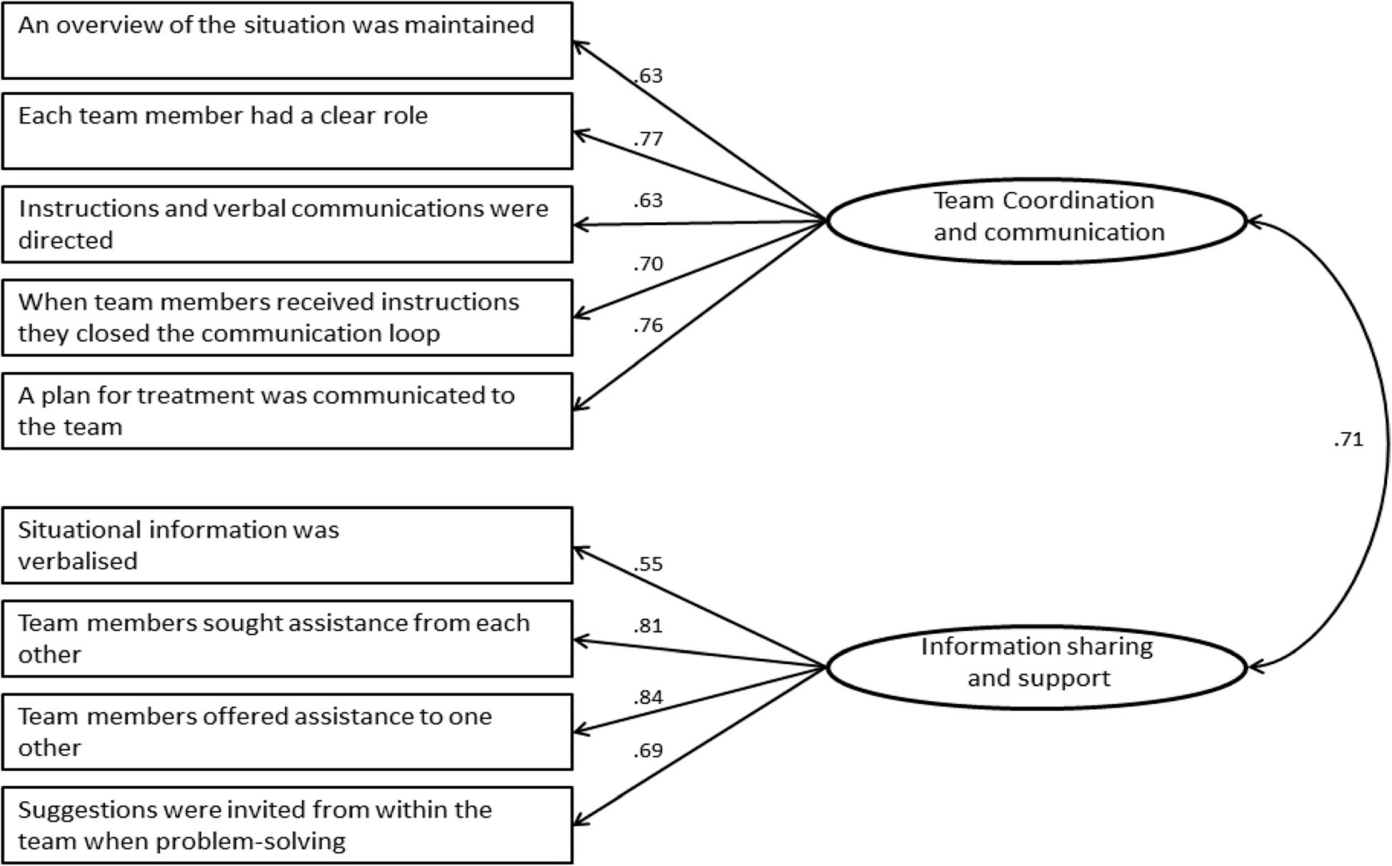
Confirmatory Factor Analysis model. Factor loadings are reported for each item and the correlation is shown between the factors

Confirmatory factor analysis was performed using the 9 items identified from the EFA (Fig. 1). The overall Goodness of Fit model was deemed acceptable (*χ*^2^ min/df = 1.768 (*p* = 0.009); GFI = 0.970; RMSEA = 0.062; CFI = .970; TLI = .948) [26]. Despite the chi-square goodness of fit being above 1.0, the GFI, RMSEA, CFI and TLI all indicated a satisfactory 2-factor solution fit.

## Discussion

The SATTS was shown to have good reliability and construct validity following analysis using EFA and CFA in the context of a large-scale mass casualty simulation which provided challenging teamwork situations that required students to work collaboratively [21].

The EFA revealed 2 factors in the SATTS which related to the constructs, *team coordination and communication* and *information sharing and support*. These are different from Weller et al. [19] which had 3 factors relating to leadership and team coordination, shared situational information, and mutual performance monitoring. The difference in factors is understandable following deliberate removal of items from the *leadership and mutual performance monitoring* construct. However, the CFA model validated these findings, as the goodness of fit model for a 2-factor solution was suitable. These results demonstrate that the SATTS is a valid tool that measures teamwork, specifically for student cohorts.

There were six items that were cross-loaded on the two factors and not included in the CFA model (see italicized items from Table 1). Four of the items (no. 3, 7, 10, and 11) were from the Weller et al*.* [19] tool, and the remaining 2 were new items developed by the simulation experts. The EFA and CFA findings provide evidence that removal of the Weller et al*.* tool items was appropriate for the student cohort. The mass casualty simulation was a complex, challenging teamwork situation and some of the items relevant to critical care teams were not suitable for undergraduate health professional students. Student teams were not experienced like existing health professional teams who normally work together. Further, the mix of clinical skills, expertise and leadership behaviours appropriate for real teams did not fit the study scenario teamwork context. Nevertheless, two of the new items related to *teamwork communication* (no. 8 and 9) did not fit the 2-factor solution. These 6 items had similar factor loadings for the 2 factors, indicating that they were not distinguishable between the *Teamwork coordination and communication* and *Information sharing and support* constructs and were correctly removed.

Teamwork tools mainly use behavioural rating scales or self-assessment formats to evaluate teamwork performance [7, 29]. While behavioural rating scales require a trained observer(s) to measure teamwork and score team performance, self-assessment tools enable users to gain insights into their own thinking and the reasons behind observed behaviours [7]. The SATTS demonstrated evidence of reliability and validity when used by students, and could potentially promote formative self-assessment of teamwork prior to facilitator-led debriefing. This tool may also enhance the educational value of future teamwork-focused simulation for students, especially when large student numbers limit the availability of small group debriefing by expert facilitators. Use of the SATTS prior to debriefing may facilitate students’ critical self-assessment of teamwork. The SATTS could function as a structured guide to reflect on the teamwork behaviours experienced by the students following simulation scenarios and potentially promote the learning process. This could potentially reduce the need for an expert to facilitate the debrief [30]. However, further research is required to establish this paradigm.

Whilst the SATTS was developed for self-assessment of teamwork in student cohorts, the context of the tool needs to be considered. The original Weller tool was designed to measure teamwork behaviours in critical care health professionals (doctors and nurses) who work in these settings. The authors chose to adapt this for undergraduate health professional students as the tool contained items indicating requisite behaviours for optimal teamwork. The use of the SATTS following the mass casualty simulation was likely to have influenced the student responses as the clinical scenario was one that had not been encountered by students previously. Therefore, it was appropriate to adapt the tool for both student teamwork and the simulation environment. Further, the validation of the SATTS using both EFA and CFA provided construct validity for teamwork communication and information sharing which are similar to other tools [17, 28].

This tool could potentially be used in other contexts, where students are exposed to different forms of teamwork training. The SATTS could be further developed for use by expert observer raters who assess teamwork performance, similar to the original critical care tool development. The tool could then be used to assess student teamwork in assessment formats, such as Team Objective Structured Clinical Examinations.

### Limitations

Whilst this study provides evidence on the validity and reliability of the SATTS tool when used by students for self-assessment, self-assessed scores tend to be more lenient than external ratings. We have not compared self-assessed scores against those of external raters or any other standard, but this could be an area for further research. Whilst there is evidence of validity of this teamwork self-assessment tool in the context of mass casualty simulations, the extent to which is it valid in other contexts remains to be established.

## Conclusion

To date, most teamwork tools focus on practising health professionals with few designed specifically for undergraduate students. The authors modified an existing self-assessment teamwork tool (developed for critical care teams) for use with undergraduate health professional medical and nursing students. The results of the present study provide evidence to support the validity and reliability of the SATTS for self-assessment of teamwork in the context of simulated mass casualties. The SATTS could be used by students to promote learning of important teamwork behaviours and potentially well suited for large scale interprofessional student teamwork activities.

## Additional file

Additional file 1: Self-assessment teamwork tool for students (SATTS) questionnaire (DOC 70 kb)

## Abbreviations

EFA: Exploratory factor analysis
CFA: Confirmatory factor analysis
SATTS: Self-assessment teamwork tool for students
