# Supplementary material for: Development of a self-assessment teamwork tool for use by medical and nursing students
Source: BMC Med Educ. 2016 Aug 24;16(1):218. doi: 10.1186/s12909-016-0743-9 (PMC4995823; doi:10.1186/s12909-016-0743-9)
Supplement: Additional file 1: — Self-assessment teamwork tool for students (SATTS) questionnaire (DOC 70 kb) [file 12909_2016_743_MOESM1_ESM.doc]

**SELF-ASSESSMENT TEAMWORK TOOL FOR STUDENTS (SATTS)**

| **Factor 1: Teamwork coordination and communication** | **Poor** |  |  | **Average** |  |  | **Excellent** | **Description** |
| --- | --- | --- | --- | --- | --- | --- | --- | --- |
| **Each team member had a clear role** | 1 | 2 | 3 | 4 | 5 | 6 | 7 | **Excellent:** All required roles were taken on. Team members clearly took ownership of particular roles, articulated their roles. No duplication or confusion over roles was evident. Team members continued with their roles as long as necessary, or until completed.  **Average**: It was generally clear what each person’s role was, and on most occasions, the required roles were covered.  **Poor**: No designation of roles occurred. Some roles were unassigned. It was unclear what team members should be doing. |
| **A plan for treatment was communicated to the team** | 1 | 2 | 3 | 4 | 5 | 6 | 7 | **Excellent**: The relevant team members were informed of a plan in sufficient detail for them to understand what was required of them.  **Average**: A general idea of a plan was communicated but detail was lacking.  **Poor**: No treatment plan was shared. |
| **When team members received instructions they closed the communication loop** | 1 | 2 | 3 | 4 | 5 | 6 | 7 | **Excellent**: For critical instructions, team members repeated back the instruction to confirm that it had been heard correctly and would be acted upon. For routine or non-critical instructions, they said or did something to indicate they’d heard.  **Poor**: No acknowledgement that the instruction had been heard or would be acted upon |
| **Instructions and verbal communications were directed.** | 1 | 2 | 3 | 4 | 5 | 6 | 7 | **Excellent**: Use of person’s name (not just directed non-verbally)  **Average**: Generally it was clear who the communication was directed at, names not used all the time, the occasional use of “someone” or undirected communication.  **Poor**: Use of “someone”, directed to the room, no non-verbal indication of who the communication was meant for. |
| **An overview of the situation was maintained** | 1 | 2 | 3 | 4 | 5 | 6 | 7 | **Excellent**: Throughout the case, at least one member of the team kept an overview of the situation (was aware of all the information available, and how tasks were being implemented, e.g. looking around at what others were doing, etc.).  **Average**: On the whole, at least one member of the team seemed to be aware of most of the available information, only occasionally getting distracted.  **Poor:** Examples – team members were slow to notice and share new information, failed to notice that some tasks weren’t being done. |
| **Factor 2: Information sharing and support** | **Poor** |  |  | **Average** |  |  | **Excellent** | **Description** |
| **Suggestions were invited from within the team when problem-solving.** | 1 | 2 | 3 | 4 | 5 | 6 | 7 | **Excellent**: Team members asked for suggestions about possible other causes or strategies to manage a problem. E.g. I think this is due to blood loss does anybody have any other ideas? Specifically, the whole team is engaged for possible information and solutions.  **Average**: See note below.  **Poor**: No opinions or suggestions are asked for, even when this would have potentially helped the situation. |
| **Team members offered assistance to one other.** | 1 | 2 | 3 | 4 | 5 | 6 | 7 | **Excellent**: Team members explicitly offered to help with tasks, with the purpose of making sure everything got done in a timely fashion.  **Average**: A few instances of team members offering assistance, some missed opportunities.  **Poor**: Team members never offered to help when it was clear others had too much to do. |
| **Team members offered assistance to one other.** | 1 | 2 | 3 | 4 | 5 | 6 | 7 | **Excellent**: Team members explicitly offered to help with tasks, with the purpose of making sure everything got done in a timely fashion.  **Average**: A few instances of team members offering assistance, some missed opportunities.  **Poor**: Team members never offered to help when it was clear others had too much to do. |
| **Situational information was verbalised** | 1 | 2 | 3 | 4 | 5 | 6 | 7 | **Excellent:** Team members verbalised patient information, situation information, monitor data or other information.  **Average:** All team members verbalised situational information occasionally. Occasional essential opportunities missed. Distracting/unnecessary communication may occur occasionally.  **Poor**: Team members didn’t say anything when there was a situation which may have needed action. E.g. no other team member saying the BP is low. |
| **Cross-loaded items** |  |  |  |  |  |  |  |  |
| **Instructions were explicit** | 1 | 2 | 3 | 4 | 5 | 6 | 7 | **Excellent**: Instructions were clearly audible, easy to understand, and there was sufficient detail (e.g. dose/ dilution of drug) to avoid any potential confusion.  **Average**: Most instructions were clear, but with the occasional vague instructions.  **Poor**: Unclear, inaudible, or imprecise instructions |
| **Priorities and orders of actions were communicated to the team** | 1 | 2 | 3 | 4 | 5 | 6 | 7 | **Excellent**: When more than one task / action was needed at any time, it was clear and explicit what was most important, and which needed to be done first.  **Average**: It reasonably clear what was most important, and what the team member(s) needed to do next.  **Poor**: There were multiple requests for actions without prioritising such that it was unclear if team members knew what order to carry out the tasks. |
| **Possible future developments or requirements were communicated clearly.** | 1 | 2 | 3 | 4 | 5 | 6 | 7 | **Excellent**: Following the initial plan, or following a change in patient status, anticipated events and what might be required were verbalised clearly.  **Average**: There was some anticipation of future developments but with insufficient detail for team members to know what may be required of them.  **Poor**: There was no information communicated about possible developments to prepare for. |
| **Questions, input, or requests for clarification were responded to appropriately.** | 1 | 2 | 3 | 4 | 5 | 6 | 7 | **Excellent**: There was a climate of inviting and respecting input. Questions were answered, concerns acknowledged, and explanation or clarification given in response to questioning.  **Poor**: Input not invited and actively inhibited. Questions or concerns were ignored or dismissed. |
| **When expressions of concern were raised and not responded to appropriately, team members persisted in seeking a response, or took action** | 1 | 2 | 3 | 4 | 5 | 6 | 7 | **Did this occur? Yes / No**  If initial concerns were always appropriately responded to, then this is No. If no concerns were ever raised, this is also No. (score N/A). **If yes:**  **Excellent**: The team member, after having their initial concern ignored, raised the issue again until the issue was resolved.  **Poor**: The team member gave up and didn’t pursue their concern, or persisted with low level probing and alerting statements in a ‘hint & hope’ fashion and the issue was never resolved. |
| **Important clinical actions were verbalised** | 1 | 2 | 3 | 4 | 5 | 6 | 7 | **Excellent**: All important actions were verbalised as they were being carried out or immediately after, e.g. injury found.  **Average:** In general, there was information shared about important actions or events.  **Poor**: It was rare for information about actions or events to be shared |
| **Removed from analysis** |  |  |  |  |  |  |  |  |
| **When faced with a problem, external assistance was sought** | 1 | 2 | 3 | 4 | 5 | 6 | 7 | **Excellent:** External advice / help was sourced in a timely way.  **Average:** Help is called for only when team is becoming overwhelmed  **Poor:** External help was never called even though this could have helped resolve the situation. |
| **A situation update was given when the situation changed** | 1 | 2 | 3 | 4 | 5 | 6 | 7 | **Excellent:** A team member described patient status, what they think is going on and/or new plans.  **Average:** A team member makes some attempt but it was unsystematic or incomplete or the information was only shared between half the team.  **Poor:** No information shared about any relevant changes or new information in patient status |
| **Overall teamwork** | 1 | 2 | 3 | 4 | 5 | 6 | 7 | **Excellent:** Overall, this was an effective and efficient team, with a great communication climate, who worked well together.  **Poor:** Overall, this was a disorganised team who worked poorly together. |
